# Poly[[μ_3_-3-(3-pyrid­yl)acrylato-κ^3^ *N*:*O*:*O*′][μ_2_-3-(3-pyrid­yl)acrylato-κ^3^ *O*,*O*′:*O*][μ_2_-3-(3-pyrid­yl)acrylato-κ^2^ *O*:*O*′)]gadolinium(III)]

**DOI:** 10.1107/S1600536808012270

**Published:** 2008-05-03

**Authors:** Zhi-Hui Qiu, Fu-Pei Liang, Qing-Feng Ruan, Zi-Lu Chen

**Affiliations:** a College of Chemistry and Chemical Engineering, Guangxi Normal University, Guilin 541004, People’s Republic of China; b Faculty of Earth Sciences, China University of Geosciences, Wuhan 430074, People’s Republic of China; c Department of Resources and Environmental Engineering, Guilin University of Technology, Guilin,541004, People’s Republic of China

## Abstract

In the title compound, [Gd(C_8_H_6_NO_2_)_3_]_*n*_, the Gd^III^ ion is in a bicapped trigonal prismatic coordination environment formed by seven O atoms and one N atom, derived from seven different 3-(3-pyrid­yl)acrylate (3-PYA) ligands. Gd^III^ ions are bridged by bidentate and tridentate 3-PYA ligands, resulting in a two-dimensional structure.

## Related literature

For related literature, see: Ayyappan *et al.* (2001 ▶); Gunning & Cahill (2005 ▶); Zhang *et al.* (2000 ▶) Liu *et al.* (2006 ▶); Liu *et al.* (2004 ▶); Zhou *et al.* (2004 ▶); Li *et al.* (2007 ▶). For related structures, see: Zhou *et al.*, (2003 ▶).
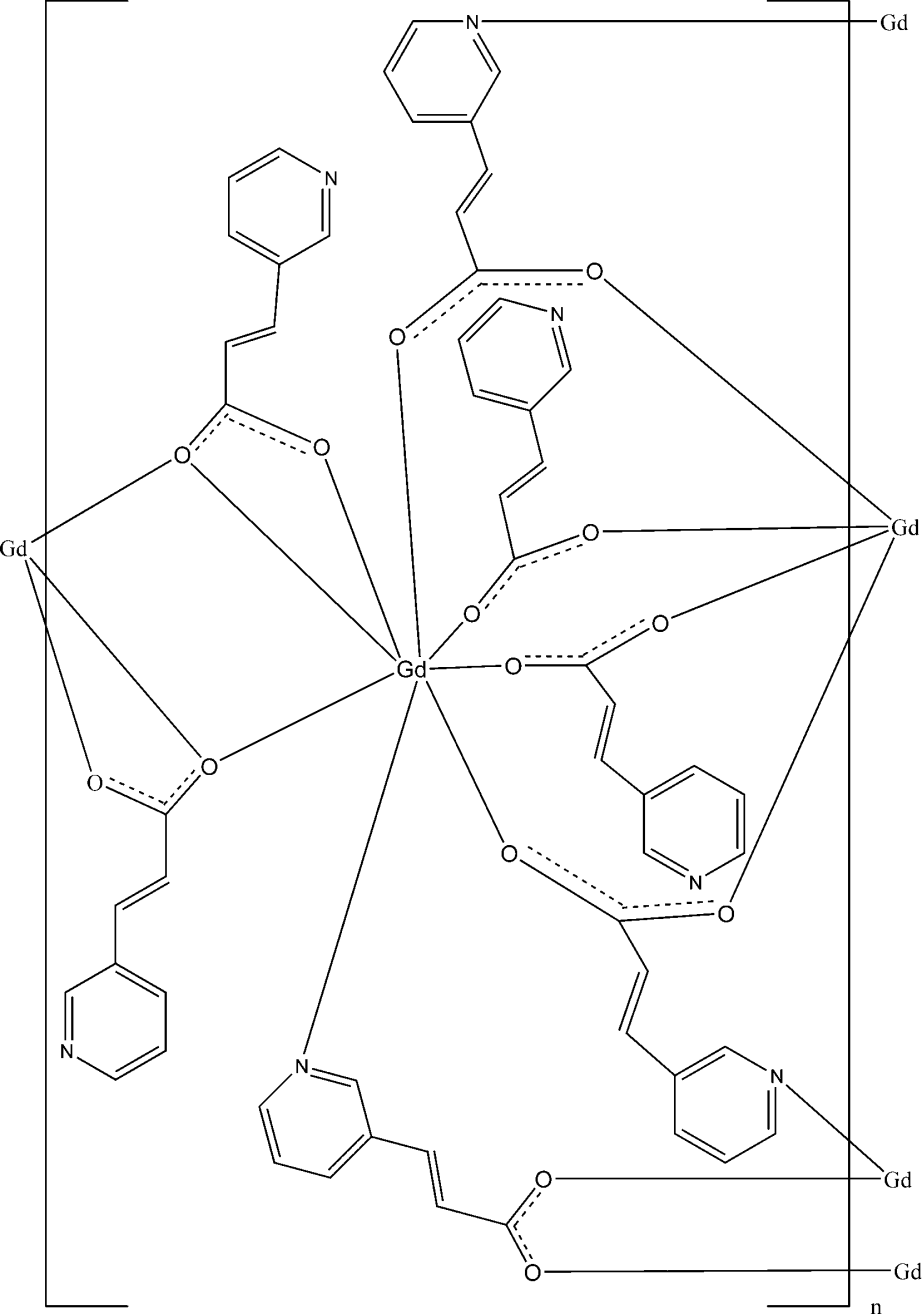


## Experimental

### 

#### Crystal data

[Gd(C_8_H_6_NO_2_)_3_]
*M* *_r_* = 601.66
Monoclinic, 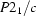

*a* = 7.7197 (17) Å
*b* = 25.646 (6) Å
*c* = 11.445 (2) Å
β = 95.684 (3)°
*V* = 2254.8 (8) Å^3^
*Z* = 4
Mo *K*α radiation
μ = 2.99 mm^−1^
*T* = 294 (2) K
0.24 × 0.20 × 0.18 mm

#### Data collection

Bruker SMART CCD diffractometer
Absorption correction: multi-scan (*SADABS*; Bruker, 1998 ▶) *T*_min_ = 0.534, *T*_max_ = 0.615 (expected range = 0.507–0.584)
12572 measured reflections
4654 independent reflections
3517 reflections with *I* > 2σ(*I*)
*R*_int_ = 0.048

#### Refinement

*R*[*F* ^2^ > 2σ(*F* ^2^)] = 0.037
*wR*(*F* ^2^) = 0.090
*S* = 1.05
4654 reflections
307 parameters
H-atom parameters constrained
Δρ_max_ = 2.06 e Å^−3^
Δρ_min_ = −1.17 e Å^−3^

### 

Data collection: *SMART* (Bruker, 1998 ▶); cell refinement: *SAINT* (Bruker, 1998 ▶); data reduction: *SAINT*; program(s) used to solve structure: *SHELXS97* (Sheldrick, 2008 ▶); program(s) used to refine structure: *SHELXL97* (Sheldrick, 2008 ▶); molecular graphics: *SHELXTL* (Sheldrick, 2008 ▶); software used to prepare material for publication: *SHELXTL*.

## Supplementary Material

Crystal structure: contains datablocks I, global. DOI: 10.1107/S1600536808012270/lh2617sup1.cif

Structure factors: contains datablocks I. DOI: 10.1107/S1600536808012270/lh2617Isup2.hkl

Additional supplementary materials:  crystallographic information; 3D view; checkCIF report

## Figures and Tables

**Table 1:** Selected bond lengths (Å)

Gd1—O4^i^	2.305 (3)
Gd1—O2^i^	2.305 (3)
Gd1—O1	2.332 (3)
Gd1—O3	2.353 (3)
Gd1—O6^ii^	2.383 (3)
Gd1—O5	2.440 (3)
Gd1—O6	2.546 (3)
Gd1—N1^iii^	2.628 (4)

Symmetry codes: (i) 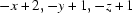; (ii) 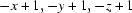; (iii) 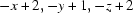.

**Fig. 1.**
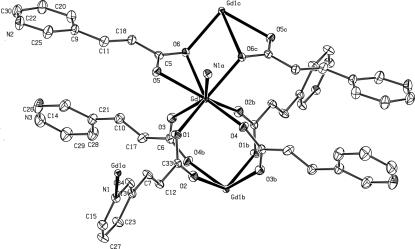
A portion of the structure of (I) showing the coordination environment of the GdIII ion, with displacement ellipsoids at the 30% probability level. All H atoms are omitted for clarity. [Symmetry codes:(a)-x,1 - y,-z;(b)-x,1 - y,1 - z;(c)1 - x,1 - y,1 - z.]

**Fig. 2.**
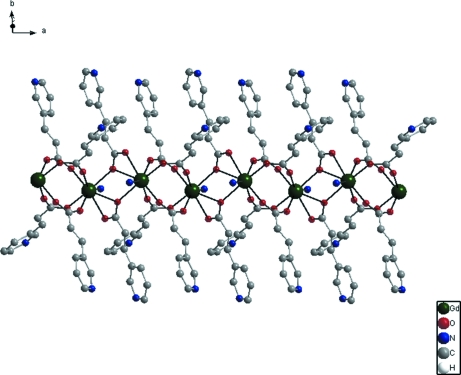
Part of a chain structure of (I), along the a axis. All H atoms are omitted.

**Fig. 3.**
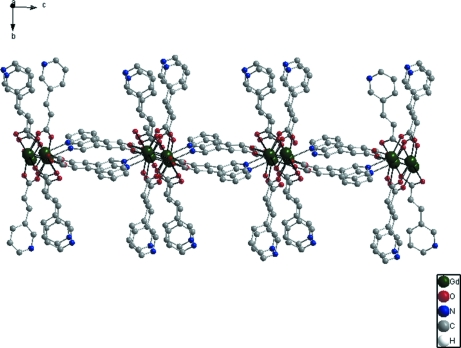
The two-dimensional structure of (I) parallel to (100), All H atoms have been omitted for clearity.

## supplementary crystallographic information

### Comment

The bifunctional compound 3-pyridylacrylic acid (3-HPYA) is a potential multidentate ligand, and several types of complexes formed with 3-HPYA have been studied (Ayyappan *et al.*, 2001; Gunning & Cahill, 2005; Zhang *et al.*, 2000). Until now, however, only a few crystallographic studies of 4*f*-block metal complexes of HPYA have been reported (Liu *et al.*,2006; Liu *et al.*,2004; Zhou *et al.*,2004; Zhou *et al.*,2003; Li *et al.*, 2007).

Here, we report the synthesis and structure of the title complex, [Gd(TPA)_3_]_n_ (I) (Fig.1). The Gd^III^ ion is eight-coordinated by seven O atoms and one N atom derived from seven different 3-PYA ligands. The topology of (I) is a two-dimensional structure mediated by bridging 3-PYA ligands. Symmetry-related Gd^III^ centres are bridged by two bidentate and two tridentate 3-PYA ligands, which results in the formation of a one-dimensional chain along *a* axis (Fig.2). Different chains are connected by tridentate 3-PYA ligands, which results in the formation of a two-dimensional framework parallel to (100) (Fig.3). Gd—O distances are in the range 2.305 (3) to 2.546 (3) Å, and the Gd—N distance is 2.628 (4) Å.

### Experimental

A mixture of Gd_2_O_3_(0.5 mmol), 3-pyridylacrylic acid (2.0 mmol), H_2_O(14 ml) was sealed in a 25 ml Teflon-lined stainless reactor and heated at 468 K for six days under autogenous pressure, then followed by slow cooling to room temperature, when a few colourless crystals were obtained. Analysis:found C 47.41,H 3.08,*N* 7.03%; C_24_H_20_GdN_3_O_7_ requires C 47.45,H 2.97,*N* 6.92%.

### Refinement

H atoms bonded were placed at calulated posotions and treated using a riding-model approximation [C—H = 0.93Å and *U*_iso_(H)= 1.2*U*_eq_(C)].

### Crystal data

**Table:**

[Gd(C_8_H_6_NO_2_)_3_]	*F*_000_ = 1180
*M**_r_* = 601.66	*D*_x_ = 1.772 Mg m^−^^3^
Monoclinic, *P*2_1_/*c*	Mo *K*α radiation λ = 0.71073 Å
Hall symbol: -P 2ybc	Cell parameters from 5417 reflections
*a* = 7.7197 (17) Å	θ = 2.7–26.5º
*b* = 25.646 (6) Å	µ = 2.99 mm^−^^1^
*c* = 11.445 (2) Å	*T* = 294 (2) K
β = 95.684 (3)º	Block, colourless
*V* = 2254.8 (8) Å^3^	0.24 × 0.20 × 0.18 mm
*Z* = 4	

### Data collection

**Table:**

Bruker SMART CCD diffractometer	4654 independent reflections
Radiation source: fine-focus sealed tube	3517 reflections with *I* > 2σ(*I*)
Monochromator: graphite	*R*_int_ = 0.048
*T* = 294(2) K	θ_max_ = 26.6º
φ and ω scans	θ_min_ = 1.6º
Absorption correction: multi-scan(SADABS; Bruker, 1998)	*h* = −9→9
*T*_min_ = 0.534, *T*_max_ = 0.615	*k* = −30→32
12572 measured reflections	*l* = −14→7

### Refinement

**Table:**

Refinement on *F*^2^	Secondary atom site location: difference Fourier map
Least-squares matrix: full	Hydrogen site location: inferred from neighbouring sites
*R*[*F*^2^ > 2σ(*F*^2^)] = 0.037	H-atom parameters constrained
*wR*(*F*^2^) = 0.090	*w* = 1/[σ^2^(*F*_o_^2^) + (0.0357*P*)^2^ + 2.29*P*] where *P* = (*F*_o_^2^ + 2*F*_c_^2^)/3
*S* = 1.06	(Δ/σ)_max_ = 0.001
4654 reflections	Δρ_max_ = 2.06 e Å^−^^3^
307 parameters	Δρ_min_ = −1.16 e Å^−^^3^
Primary atom site location: structure-invariant direct methods	Extinction correction: none

### Special details

**Table:**

Geometry. All e.s.d.'s (except the e.s.d. in the dihedral angle between two l.s. planes) are estimated using the full covariance matrix. The cell e.s.d.'s are taken into account individually in the estimation of e.s.d.'s in distances, angles and torsion angles; correlations between e.s.d.'s in cell parameters are only used when they are defined by crystal symmetry. An approximate (isotropic) treatment of cell e.s.d.'s is used for estimating e.s.d.'s involving l.s. planes.
Refinement. Refinement of *F*^2^ against ALL reflections. The weighted *R*-factor *wR* and goodness of fit *S* are based on *F*^2^, conventional *R*-factors *R* are based on *F*, with *F* set to zero for negative *F*^2^. The threshold expression of *F*^2^ > σ(*F*^2^) is used only for calculating *R*-factors(gt) *etc*. and is not relevant to the choice of reflections for refinement. *R*-factors based on *F*^2^ are statistically about twice as large as those based on *F*, and *R*- factors based on ALL data will be even larger.

### Fractional atomic coordinates and isotropic or equivalent isotropic displacement parameters (Å^2^)

**Table:**

	*x*	*y*	*z*	*U*_iso_*/*U*_eq_	
Gd1	0.75345 (2)	0.501980 (8)	0.574129 (16)	0.01502 (9)	
O1	0.9665 (4)	0.52846 (14)	0.7208 (3)	0.0273 (7)	
O2	1.1908 (5)	0.52067 (14)	0.6134 (3)	0.0311 (8)	
O3	0.8511 (4)	0.58040 (13)	0.4964 (3)	0.0306 (8)	
O4	1.0842 (4)	0.57303 (13)	0.3946 (3)	0.0302 (8)	
O5	0.6072 (4)	0.57155 (13)	0.6716 (3)	0.0262 (7)	
O6	0.4848 (4)	0.55290 (13)	0.4935 (2)	0.0236 (7)	
N1	1.3676 (5)	0.53963 (15)	1.2405 (3)	0.0240 (9)	
C5	0.5075 (6)	0.58425 (18)	0.5821 (4)	0.0219 (10)	
C6	0.9606 (6)	0.59796 (18)	0.4321 (4)	0.0236 (10)	
C7	1.2073 (6)	0.5432 (2)	0.9203 (4)	0.0275 (11)	
H7	1.0910	0.5364	0.9292	0.033*	
C9	0.3667 (7)	0.72221 (19)	0.6627 (4)	0.0298 (11)	
C10	0.8451 (7)	0.6892 (2)	0.4348 (4)	0.0330 (12)	
H10	0.7659	0.6781	0.4857	0.040*	
C11	0.4341 (7)	0.6688 (2)	0.6617 (4)	0.0314 (12)	
H11	0.4939	0.6570	0.7312	0.038*	
C12	1.2511 (6)	0.54388 (19)	0.8113 (4)	0.0245 (10)	
H12	1.3640	0.5534	0.7986	0.029*	
C13	1.3224 (6)	0.55200 (19)	1.0284 (4)	0.0248 (10)	
C14	0.7726 (8)	0.7816 (2)	0.4738 (6)	0.0453 (15)	
H14	0.7220	0.7695	0.5390	0.054*	
C15	1.5195 (6)	0.5655 (2)	1.2392 (4)	0.0288 (11)	
H15	1.5874	0.5703	1.3101	0.035*	
N2	0.3364 (7)	0.80038 (19)	0.7784 (4)	0.0468 (13)	
C17	0.9503 (7)	0.6539 (2)	0.3963 (5)	0.0347 (13)	
H17	1.0246	0.6649	0.3420	0.042*	
C18	0.4192 (6)	0.6355 (2)	0.5732 (4)	0.0290 (11)	
H18	0.3521	0.6444	0.5041	0.035*	
C20	0.2839 (7)	0.7472 (2)	0.5650 (4)	0.0370 (13)	
H20	0.2652	0.7298	0.4937	0.044*	
C21	0.8437 (7)	0.7449 (2)	0.4033 (5)	0.0332 (12)	
C22	0.2294 (8)	0.7983 (2)	0.5747 (5)	0.0447 (15)	
H22	0.1733	0.8155	0.5102	0.054*	
C23	1.4821 (6)	0.5782 (2)	1.0315 (4)	0.0309 (12)	
H23	1.5217	0.5907	0.9627	0.037*	
N3	0.7710 (7)	0.8333 (2)	0.4554 (5)	0.0582 (15)	
C25	0.3879 (8)	0.7510 (2)	0.7660 (5)	0.0425 (14)	
H25	0.4425	0.7345	0.8321	0.051*	
C26	0.8422 (8)	0.8500 (2)	0.3614 (6)	0.0539 (17)	
H26	0.8422	0.8856	0.3465	0.065*	
C27	1.5802 (6)	0.5853 (2)	1.1382 (4)	0.0326 (12)	
H27	1.6855	0.6031	1.1421	0.039*	
C28	0.9162 (8)	0.7645 (2)	0.3054 (5)	0.0429 (14)	
H28	0.9645	0.7418	0.2542	0.052*	
C29	0.9163 (9)	0.8174 (2)	0.2846 (6)	0.0515 (16)	
H29	0.9653	0.8308	0.2200	0.062*	
C30	0.2599 (8)	0.8233 (2)	0.6821 (5)	0.0441 (15)	
H30	0.2253	0.8578	0.6873	0.053*	
C33	1.1260 (6)	0.52998 (18)	0.7078 (4)	0.0203 (9)	
C34	1.2735 (6)	0.53395 (19)	1.1353 (4)	0.0272 (11)	
H34	1.1676	0.5166	1.1340	0.033*	

### Atomic displacement parameters (Å^2^)

**Table:**

	*U*^11^	*U*^22^	*U*^33^	*U*^12^	*U*^13^	*U*^23^
Gd1	0.01666 (12)	0.01601 (13)	0.01255 (12)	−0.00038 (9)	0.00230 (8)	−0.00069 (9)
O1	0.0229 (17)	0.036 (2)	0.0223 (17)	−0.0025 (15)	−0.0008 (14)	−0.0003 (14)
O2	0.038 (2)	0.036 (2)	0.0197 (17)	−0.0029 (16)	0.0062 (15)	−0.0066 (15)
O3	0.0317 (19)	0.0241 (19)	0.038 (2)	−0.0019 (15)	0.0136 (16)	0.0060 (15)
O4	0.0315 (19)	0.0218 (18)	0.038 (2)	0.0080 (15)	0.0080 (16)	0.0036 (15)
O5	0.0279 (18)	0.0274 (19)	0.0230 (17)	0.0059 (14)	0.0011 (14)	−0.0011 (14)
O6	0.0245 (17)	0.0224 (17)	0.0241 (17)	−0.0025 (13)	0.0027 (14)	−0.0061 (13)
N1	0.026 (2)	0.024 (2)	0.021 (2)	−0.0016 (17)	−0.0005 (16)	0.0013 (16)
C5	0.022 (2)	0.021 (2)	0.022 (2)	−0.0032 (19)	0.0024 (19)	0.0027 (19)
C6	0.028 (3)	0.019 (2)	0.023 (2)	−0.0021 (19)	0.000 (2)	0.0005 (18)
C7	0.026 (2)	0.036 (3)	0.020 (2)	−0.011 (2)	−0.0020 (19)	−0.001 (2)
C9	0.035 (3)	0.027 (3)	0.028 (3)	0.003 (2)	0.005 (2)	−0.007 (2)
C10	0.036 (3)	0.027 (3)	0.037 (3)	0.003 (2)	0.009 (2)	0.004 (2)
C11	0.037 (3)	0.031 (3)	0.025 (3)	0.007 (2)	0.001 (2)	0.000 (2)
C12	0.023 (2)	0.032 (3)	0.019 (2)	−0.007 (2)	0.0044 (19)	−0.0048 (19)
C13	0.029 (3)	0.026 (3)	0.020 (2)	−0.005 (2)	0.003 (2)	−0.0030 (19)
C14	0.047 (4)	0.036 (3)	0.054 (4)	0.012 (3)	0.008 (3)	0.004 (3)
C15	0.030 (3)	0.032 (3)	0.023 (3)	0.000 (2)	−0.004 (2)	0.003 (2)
N2	0.064 (3)	0.033 (3)	0.043 (3)	0.007 (2)	0.003 (3)	−0.012 (2)
C17	0.033 (3)	0.029 (3)	0.046 (3)	0.007 (2)	0.017 (2)	0.013 (2)
C18	0.030 (3)	0.027 (3)	0.029 (3)	0.003 (2)	0.000 (2)	−0.004 (2)
C20	0.048 (3)	0.029 (3)	0.033 (3)	0.004 (2)	−0.001 (3)	−0.003 (2)
C21	0.037 (3)	0.026 (3)	0.036 (3)	0.006 (2)	0.003 (2)	0.004 (2)
C22	0.059 (4)	0.026 (3)	0.048 (4)	0.007 (3)	0.002 (3)	0.004 (3)
C23	0.032 (3)	0.038 (3)	0.024 (2)	−0.011 (2)	0.004 (2)	0.003 (2)
N3	0.068 (4)	0.026 (3)	0.081 (4)	0.013 (3)	0.009 (3)	−0.004 (3)
C25	0.053 (4)	0.036 (3)	0.036 (3)	0.013 (3)	−0.004 (3)	−0.005 (2)
C26	0.059 (4)	0.024 (3)	0.075 (5)	0.007 (3)	−0.014 (4)	0.008 (3)
C27	0.028 (3)	0.040 (3)	0.030 (3)	−0.011 (2)	0.000 (2)	0.003 (2)
C28	0.057 (4)	0.032 (3)	0.041 (3)	0.008 (3)	0.013 (3)	0.006 (2)
C29	0.065 (4)	0.033 (3)	0.057 (4)	0.005 (3)	0.008 (3)	0.016 (3)
C30	0.052 (4)	0.023 (3)	0.059 (4)	0.004 (3)	0.013 (3)	0.000 (3)
C33	0.024 (2)	0.021 (2)	0.016 (2)	−0.0032 (19)	0.0031 (18)	−0.0010 (17)
C34	0.028 (3)	0.030 (3)	0.023 (2)	−0.011 (2)	0.000 (2)	−0.002 (2)

### Geometric parameters (Å, °)

**Table:**

Gd1—O4^i^	2.305 (3)	C11—C18	1.321 (6)
Gd1—O2^i^	2.305 (3)	C11—H11	0.9300
Gd1—O1	2.332 (3)	C12—C33	1.495 (6)
Gd1—O3	2.353 (3)	C12—H12	0.9300
Gd1—O6^ii^	2.383 (3)	C13—C34	1.394 (6)
Gd1—O5	2.440 (3)	C13—C23	1.401 (6)
Gd1—O6	2.546 (3)	C14—N3	1.343 (8)
Gd1—N1^iii^	2.628 (4)	C14—C21	1.389 (8)
Gd1—C5	2.846 (5)	C14—H14	0.9300
O1—C33	1.255 (5)	C15—C27	1.385 (7)
O2—C33	1.257 (5)	C15—H15	0.9300
O2—Gd1^i^	2.305 (3)	N2—C30	1.333 (7)
O3—C6	1.258 (5)	N2—C25	1.340 (7)
O4—C6	1.259 (6)	C17—H17	0.9300
O4—Gd1^i^	2.305 (3)	C18—H18	0.9300
O5—C5	1.262 (5)	C20—C22	1.383 (8)
O6—C5	1.293 (5)	C20—H20	0.9300
O6—Gd1^ii^	2.383 (3)	C21—C28	1.395 (7)
N1—C15	1.348 (6)	C22—C30	1.386 (8)
N1—C34	1.351 (6)	C22—H22	0.9300
N1—Gd1^iii^	2.628 (4)	C23—C27	1.383 (7)
C5—C18	1.479 (7)	C23—H23	0.9300
C6—C17	1.492 (7)	N3—C26	1.326 (8)
C7—C12	1.325 (6)	C25—H25	0.9300
C7—C13	1.468 (6)	C26—C29	1.378 (9)
C7—H7	0.9300	C26—H26	0.9300
C9—C20	1.388 (7)	C27—H27	0.9300
C9—C25	1.389 (7)	C28—C29	1.377 (8)
C9—C11	1.466 (7)	C28—H28	0.9300
C10—C17	1.321 (7)	C29—H29	0.9300
C10—C21	1.472 (7)	C30—H30	0.9300
C10—H10	0.9300	C34—H34	0.9300
			
O4^i^—Gd1—O2^i^	77.61 (12)	C17—C10—C21	124.9 (5)
O4^i^—Gd1—O1	78.31 (12)	C17—C10—H10	117.5
O2^i^—Gd1—O1	124.09 (12)	C21—C10—H10	117.5
O4^i^—Gd1—O3	125.63 (12)	C18—C11—C9	127.3 (5)
O2^i^—Gd1—O3	76.54 (12)	C18—C11—H11	116.3
O1—Gd1—O3	78.10 (12)	C9—C11—H11	116.3
O4^i^—Gd1—O6^ii^	87.00 (12)	C7—C12—C33	122.5 (4)
O2^i^—Gd1—O6^ii^	76.00 (11)	C7—C12—H12	118.7
O1—Gd1—O6^ii^	150.72 (11)	C33—C12—H12	118.7
O3—Gd1—O6^ii^	130.38 (11)	C34—C13—C23	116.8 (4)
O4^i^—Gd1—O5	143.96 (11)	C34—C13—C7	119.6 (4)
O2^i^—Gd1—O5	138.40 (12)	C23—C13—C7	123.6 (4)
O1—Gd1—O5	77.47 (11)	N3—C14—C21	125.3 (6)
O3—Gd1—O5	74.21 (11)	N3—C14—H14	117.4
O6^ii^—Gd1—O5	101.73 (11)	C21—C14—H14	117.4
O4^i^—Gd1—O6	153.58 (11)	N1—C15—C27	123.7 (4)
O2^i^—Gd1—O6	90.69 (11)	N1—C15—H15	118.1
O1—Gd1—O6	127.08 (11)	C27—C15—H15	118.1
O3—Gd1—O6	72.81 (11)	C30—N2—C25	116.1 (5)
O6^ii^—Gd1—O6	67.07 (13)	C10—C17—C6	125.8 (5)
O5—Gd1—O6	52.66 (10)	C10—C17—H17	117.1
O4^i^—Gd1—N1^iii^	76.53 (12)	C6—C17—H17	117.1
O2^i^—Gd1—N1^iii^	139.77 (13)	C11—C18—C5	121.1 (5)
O1—Gd1—N1^iii^	79.57 (11)	C11—C18—H18	119.4
O3—Gd1—N1^iii^	143.62 (12)	C5—C18—H18	119.4
O6^ii^—Gd1—N1^iii^	72.39 (11)	C22—C20—C9	119.5 (5)
O5—Gd1—N1^iii^	73.15 (11)	C22—C20—H20	120.2
O6—Gd1—N1^iii^	99.15 (11)	C9—C20—H20	120.2
O4^i^—Gd1—C5	165.50 (12)	C14—C21—C28	115.8 (5)
O2^i^—Gd1—C5	113.80 (13)	C14—C21—C10	120.6 (5)
O1—Gd1—C5	100.73 (12)	C28—C21—C10	123.6 (5)
O3—Gd1—C5	67.56 (12)	C20—C22—C30	118.8 (5)
O6^ii^—Gd1—C5	87.27 (12)	C20—C22—H22	120.6
O5—Gd1—C5	26.20 (11)	C30—C22—H22	120.6
O6—Gd1—C5	27.02 (11)	C27—C23—C13	119.2 (4)
N1^iii^—Gd1—C5	89.04 (12)	C27—C23—H23	120.4
C33—O1—Gd1	123.6 (3)	C13—C23—H23	120.4
C33—O2—Gd1^i^	167.0 (3)	C26—N3—C14	116.5 (5)
C6—O3—Gd1	141.8 (3)	N2—C25—C9	125.5 (5)
C6—O4—Gd1^i^	142.2 (3)	N2—C25—H25	117.2
C5—O5—Gd1	95.2 (3)	C9—C25—H25	117.2
C5—O6—Gd1^ii^	131.5 (3)	N3—C26—C29	123.7 (6)
C5—O6—Gd1	89.5 (3)	N3—C26—H26	118.2
Gd1^ii^—O6—Gd1	112.93 (13)	C29—C26—H26	118.2
C15—N1—C34	115.8 (4)	C23—C27—C15	119.2 (5)
C15—N1—Gd1^iii^	126.0 (3)	C23—C27—H27	120.4
C34—N1—Gd1^iii^	118.2 (3)	C15—C27—H27	120.4
O5—C5—O6	120.1 (4)	C29—C28—C21	120.1 (6)
O5—C5—C18	121.7 (4)	C29—C28—H28	119.9
O6—C5—C18	118.2 (4)	C21—C28—H28	119.9
O5—C5—Gd1	58.6 (2)	C26—C29—C28	118.7 (6)
O6—C5—Gd1	63.4 (2)	C26—C29—H29	120.7
C18—C5—Gd1	163.9 (3)	C28—C29—H29	120.7
O3—C6—O4	126.4 (4)	N2—C30—C22	123.5 (5)
O3—C6—C17	119.0 (4)	N2—C30—H30	118.3
O4—C6—C17	114.6 (4)	C22—C30—H30	118.3
C12—C7—C13	127.0 (5)	O1—C33—O2	125.1 (4)
C12—C7—H7	116.5	O1—C33—C12	118.5 (4)
C13—C7—H7	116.5	O2—C33—C12	116.4 (4)
C20—C9—C25	116.5 (5)	N1—C34—C13	125.3 (4)
C20—C9—C11	124.1 (4)	N1—C34—H34	117.4
C25—C9—C11	119.4 (5)	C13—C34—H34	117.4
			
O4^i^—Gd1—O1—C33	63.2 (4)	O4^i^—Gd1—C5—C18	153.1 (10)
O2^i^—Gd1—O1—C33	−3.0 (4)	O2^i^—Gd1—C5—C18	−66.6 (12)
O3—Gd1—O1—C33	−67.5 (4)	O1—Gd1—C5—C18	68.3 (12)
O6^ii^—Gd1—O1—C33	124.6 (3)	O3—Gd1—C5—C18	−3.9 (11)
O5—Gd1—O1—C33	−143.7 (4)	O6^ii^—Gd1—C5—C18	−140.0 (12)
O6—Gd1—O1—C33	−124.9 (3)	O5—Gd1—C5—C18	96.1 (12)
N1^iii^—Gd1—O1—C33	141.4 (4)	O6—Gd1—C5—C18	−100.0 (12)
C5—Gd1—O1—C33	−131.6 (4)	N1^iii^—Gd1—C5—C18	147.5 (12)
O4^i^—Gd1—O3—C6	25.1 (5)	Gd1—O3—C6—O4	−9.9 (9)
O2^i^—Gd1—O3—C6	−38.6 (5)	Gd1—O3—C6—C17	171.4 (4)
O1—Gd1—O3—C6	91.1 (5)	Gd1^i^—O4—C6—O3	−16.9 (9)
O6^ii^—Gd1—O3—C6	−96.6 (5)	Gd1^i^—O4—C6—C17	161.9 (4)
O5—Gd1—O3—C6	171.3 (5)	C20—C9—C11—C18	−4.1 (9)
O6—Gd1—O3—C6	−133.6 (5)	C25—C9—C11—C18	177.1 (6)
N1^iii^—Gd1—O3—C6	144.4 (5)	C13—C7—C12—C33	174.8 (5)
C5—Gd1—O3—C6	−161.8 (5)	C12—C7—C13—C34	−159.3 (5)
O4^i^—Gd1—O5—C5	−159.1 (3)	C12—C7—C13—C23	20.9 (8)
O2^i^—Gd1—O5—C5	24.2 (3)	C34—N1—C15—C27	−0.4 (7)
O1—Gd1—O5—C5	152.1 (3)	Gd1^iii^—N1—C15—C27	177.8 (4)
O3—Gd1—O5—C5	71.1 (3)	C21—C10—C17—C6	176.4 (5)
O6^ii^—Gd1—O5—C5	−57.9 (3)	O3—C6—C17—C10	6.8 (8)
O6—Gd1—O5—C5	−9.1 (2)	O4—C6—C17—C10	−172.1 (5)
N1^iii^—Gd1—O5—C5	−125.2 (3)	C9—C11—C18—C5	174.6 (5)
O4^i^—Gd1—O6—C5	147.4 (3)	O5—C5—C18—C11	−3.9 (7)
O2^i^—Gd1—O6—C5	−149.8 (3)	O6—C5—C18—C11	178.5 (4)
O1—Gd1—O6—C5	−14.5 (3)	Gd1—C5—C18—C11	−89.9 (12)
O3—Gd1—O6—C5	−74.2 (2)	C25—C9—C20—C22	0.6 (8)
O6^ii^—Gd1—O6—C5	135.7 (3)	C11—C9—C20—C22	−178.2 (5)
O5—Gd1—O6—C5	8.8 (2)	N3—C14—C21—C28	−0.9 (9)
N1^iii^—Gd1—O6—C5	69.3 (3)	N3—C14—C21—C10	177.3 (6)
O4^i^—Gd1—O6—Gd1^ii^	11.7 (3)	C17—C10—C21—C14	−159.1 (6)
O2^i^—Gd1—O6—Gd1^ii^	74.46 (14)	C17—C10—C21—C28	18.9 (9)
O1—Gd1—O6—Gd1^ii^	−150.16 (12)	C9—C20—C22—C30	0.3 (9)
O3—Gd1—O6—Gd1^ii^	150.14 (15)	C34—C13—C23—C27	−0.8 (8)
O6^ii^—Gd1—O6—Gd1^ii^	0.0	C7—C13—C23—C27	179.0 (5)
O5—Gd1—O6—Gd1^ii^	−126.89 (17)	C21—C14—N3—C26	0.6 (10)
N1^iii^—Gd1—O6—Gd1^ii^	−66.37 (14)	C30—N2—C25—C9	−0.6 (9)
C5—Gd1—O6—Gd1^ii^	−135.7 (3)	C20—C9—C25—N2	−0.5 (9)
Gd1—O5—C5—O6	16.6 (4)	C11—C9—C25—N2	178.4 (6)
Gd1—O5—C5—C18	−161.1 (4)	C14—N3—C26—C29	−0.3 (10)
Gd1^ii^—O6—C5—O5	105.1 (5)	C13—C23—C27—C15	1.1 (8)
Gd1—O6—C5—O5	−15.8 (4)	N1—C15—C27—C23	−0.5 (8)
Gd1^ii^—O6—C5—C18	−77.2 (5)	C14—C21—C28—C29	0.9 (9)
Gd1—O6—C5—C18	161.9 (4)	C10—C21—C28—C29	−177.2 (6)
Gd1^ii^—O6—C5—Gd1	120.9 (3)	N3—C26—C29—C28	0.4 (10)
O4^i^—Gd1—C5—O5	57.1 (6)	C21—C28—C29—C26	−0.6 (10)
O2^i^—Gd1—C5—O5	−162.7 (2)	C25—N2—C30—C22	1.6 (9)
O1—Gd1—C5—O5	−27.7 (3)	C20—C22—C30—N2	−1.5 (9)
O3—Gd1—C5—O5	−99.9 (3)	Gd1—O1—C33—O2	4.5 (7)
O6^ii^—Gd1—C5—O5	123.9 (3)	Gd1—O1—C33—C12	−175.5 (3)
O6—Gd1—C5—O5	164.0 (4)	Gd1^i^—O2—C33—O1	−7.7 (18)
N1^iii^—Gd1—C5—O5	51.5 (3)	Gd1^i^—O2—C33—C12	172.4 (12)
O4^i^—Gd1—C5—O6	−106.9 (5)	C7—C12—C33—O1	15.5 (7)
O2^i^—Gd1—C5—O6	33.3 (3)	C7—C12—C33—O2	−164.5 (5)
O1—Gd1—C5—O6	168.3 (2)	C15—N1—C34—C13	0.7 (7)
O3—Gd1—C5—O6	96.1 (2)	Gd1^iii^—N1—C34—C13	−177.6 (4)
O6^ii^—Gd1—C5—O6	−40.1 (3)	C23—C13—C34—N1	−0.1 (8)
O5—Gd1—C5—O6	−164.0 (4)	C7—C13—C34—N1	−179.9 (5)
N1^iii^—Gd1—C5—O6	−112.5 (2)		

Symmetry codes: (i) −*x*+2, −*y*+1, −*z*+1; (ii) −*x*+1, −*y*+1, −*z*+1; (iii) −*x*+2, −*y*+1, −*z*+2.
